# A Systematic Literature Review on Traumatic Optic Neuropathy

**DOI:** 10.1155/2021/5553885

**Published:** 2021-02-26

**Authors:** Saeed Karimi, Amir Arabi, Iman Ansari, Toktam Shahraki, Sare Safi

**Affiliations:** ^1^Ophthalmic Research Center, Research Institute for Ophthalmology and Vision Science, Shahid Beheshti University of Medical Sciences, Tehran, Iran; ^2^Department of Ophthalmology, Torfeh Medical Center, Shahid Beheshti University of Medical Sciences, Tehran, Iran; ^3^Ophthalmic Epidemiology Research Center, Research Institute for Ophthalmology and Vision Science, Shahid Beheshti University of Medical Sciences, Tehran, Iran

## Abstract

Traumatic optic neuropathy (TON) is an uncommon vision-threatening disorder that can be caused by ocular or head trauma and is categorized into direct and indirect TON. The overall incidence of TON is 0.7–2.5%, and indirect TON has a higher prevalence than direct TON. Detection of an afferent pupillary defect in the presence of an intact globe in a patient with ocular or head trauma with decreased visual acuity strongly suggests TON. However, afferent pupillary defects may be difficult to detect in patients who have received narcotics that cause pupillary constriction and in those with bilateral TON. Mechanical shearing of the optic nerve axons and contusion necrosis due to immediate ischemia from damage to the optic nerve microcirculation and apoptosis of neurons is a probable mechanism. The proper management of TON is controversial. High-dose corticosteroid therapy and decompression of the optic nerve provide no additional benefit over observation alone. Intravenous erythropoietin may be a safe and efficient treatment for patients with TON.

## 1. Introduction

Traumatic optic neuropathy (TON) is a vision-threatening disorder that can be caused by either ocular or head trauma and is categorized into direct and indirect TON [1, 2]. Direct TON is frequently associated with severe visual loss and a lower chance of recovery compared to indirect TON [2]. Direct TON often occurs when the optic nerve is lacerated with bone fragments or when contusion or concussion causes anatomical disruption [2, 3]. In contrast, indirect TON often occurs when a blunt head or ocular traumatic stress is transmitted through the oculofacial soft tissues and skeleton to the optic nerve; this damages the integrity of the optic nerve, leading to mild-to-severe vision loss [2, 4, 5]. It usually occurs at the junction of the intraorbital and intracanalicular segments causing compression and disruption of the pial vessels, thereby reducing the vascular supply of the optic nerve [6, 7].

## 2. Materials and Methods

The literature published from 1950 to March 2020 was reviewed by searching the ISI Web of Knowledge database, PubMed, Scopus, Embase, and the Cochrane Library. The following keywords were used: “traumatic optic neuropathy,” “TON,” “treatment,” “direct TON,” “indirect TON,” “pathogenesis,” and “prognosis.” No language limitations were applied. Articles and meta-analyses that published information about TON were selected through review of abstracts, references, and titles.

## 3. Results

### 3.1. Epidemiology

TON is an unusual cause of visual impairment after blunt or penetrating head trauma. The overall incidence of TON is 0.7–2.5% [8–11]. Indirect TON has a higher prevalence than direct TON. It occurs in 0.5% to 5% of all patients with closed head injury and 2.5% of patients with midfacial fractures [5, 12]. Intracanalicular part is the most common site of indirect TON (71.4%), followed by the orbital apex (16.7%). Involvement of both the intracanalicular segment and orbital apex was found in 11.9% of the cases. The intracranial portion of the optic nerve adjacent to the falciform ligament is another common site for optic nerve traumatic injury [13, 14].

TON has a gender predominance. Up to 80% of patients with TON have been reported to be male with a median age of 31 years, and 21% are younger than 18 years [11, 15]. Having a fall (26%), motor vehicle accidents (21%), and assaults (21%) are common etiologies of TON in the general population. However, in trauma settings, motor vehicle accidents (63%) and falling down are the main etiologies [11, 15]. TON occurs in 0.4% of patients with any kind of trauma [15]. There is a prominent association between TON and head injury, wherein all patients with TON have head injuries (two-thirds of them have a significant head injury). However, only 2.3% of the patients with head trauma experience concomitant TON [15]. Epidemiologic features of TON in pediatric patients are similar to those in adults [16]. Having a fall (50%) and motor vehicle accidents (40%) are the most common causes of TON in the pediatric population [17].

### 3.2. Pathogenesis

The pathophysiology of TON is not yet fully understood, but several mechanisms have been proposed. TON cases can be categorized as primary or secondary. Mechanical shearing of the optic nerve axons and contusion necrosis due to immediate ischemia from damage to the optic nerve microcirculation are primary mechanisms, while apoptosis of both injured and initially intact adjacent neurons is the mainstay of secondary TON [2, 4, 18]. Many patients have the involvement of both mechanisms to a certain degree.

An essential part of the pathophysiology of indirect TON is the effect of traumatic loads on the biomechanical response of the cranial contents. One study using holographic interferometry on human skulls suggested that damage to the frontal region deforms the ipsilateral orbital roof, causing damage to the optic nerve and its supporting vasculature, especially where the nerve enters the optic canal [7]. Based on an anatomic study of cadaveric orbits and optic nerves, direct shearing injury to axons, disruption of the blood supply, and pressure from microhematomas and edema due to the damage of anastomoses running between the dura and pia are the possible mechanisms of optic nerve damage [19].

Another mechanism that is thought to be involved in TON is diffuse axonal injury. Detrimental inertial forces to the head cause diffuse axonal damage, which is associated with poor neurological outcomes. Following head injury, axons of the brain white matter become rapidly deformed, resulting in axonal cytoskeleton damage and impaired axoplasmic transmission [20].

It is necessary to improve the present knowledge about the association between the biomechanics and pathophysiology related to axonal trauma. Data from trauma modeling in a virtual head and orbit show that trauma to the frontal region, even at low intensity, transmits toward the optic foramen, resulting in indirect TON. This finding supports the theory that indirect TON is more likely to be caused by a facial injury, rather than trauma to the skull. Despite this progress, the mechanism of indirect TON remains unknown. A holistic model of the orbit, containing both bone and soft tissue components, will be an excellent tool to study the pathophysiology of this vision-threatening disorder and may help develop approaches for its prevention and mitigation [21].

### 3.3. Diagnosis

#### 3.3.1. Clinical Finding

In patients with craniofacial trauma and normal globe and optic nerve head appearance, any evidence of optic nerve dysfunction (reduced vision and an afferent pupillary defect) suggests the diagnosis of indirect TON [2, 4]. Clinical findings that help diagnose TON include (1) ocular injury, (2) a relative afferent pupillary defect (RAPD), (3) variable degrees of vision loss, (4) color vision disorder, and (5) different degrees of visual field defects. RAPD is a valuable finding, and in cases with mild TON, it may be the only clinical finding before overt optic nerve atrophy. The fact that RAPD is negative in bilaterally symmetric cases should be considered. Visual acuity (VA) may range from normal to no light perception, and 40–60% of cases have light perception or worse at the time of first ophthalmic visit. Although the poor VA of the patients may not allow the ophthalmologist to attain valuable results, automated visual field testing should be offered in feasible circumstances [15, 22–24].

Anatomical location and the timing of damage are factors that determine the optic disc involvement and appearance. In most cases, the posterior part of the optic nerve is damaged, and the optic disc is often normal. In cases where the optic nerve is damaged anterior to the entry site of the central retinal vessels, swelling of the optic disc and retinal hemorrhage are evident on examination of the optic nerve head. Independent of the initial appearance of the optic disc, atrophy and pallor of the optic nerve occur approximately six weeks after the initial injury [25].

#### 3.3.2. Imaging

There is a controversy about the role of neuroimaging in TON diagnosis. Some physicians prefer to perform computed tomography (CT) scan and magnetic resonance imaging (MRI) in all patients, while the others preserve imaging modalities for cases with progressive visual impairment or when therapeutic interventions are being considered [26–28]. As such, patients with head or oculofacial trauma and simultaneous symptoms of optic nerve damage (unilateral or bilateral decreased VA, visual field defect, and an afferent pupillary defect on examination) should undergo urgent radiological investigations [29].

#### 3.3.3. CT Scan

CT is the best and most accessible imaging method for detecting optic canal fractures, orbital wall fractures, and the presence of blood in the orbit. It is very helpful in diagnosing direct or indirect TON and can also be used as a guide map for surgical interventions. It is observed that in patients with indirect TON and posterior orbital fractures, the prognosis is poorer than that in patients with indirect TON and anterior orbital fractures, suggesting a prognostic value for CT scan [25, 29, 30].

#### 3.3.4. MRI

Although MRI is not as readily available as CT in trauma settings, it can be very helpful in evaluating the optic nerve integrity and detecting nerve sheath hematoma [29]. However, MRI should be avoided if a retained metallic foreign body may be suspected when trauma is occurring. Different MRI techniques are useful in the diagnosis and follow-up of patients with indirect TON. In a study by Bodanapally et al., it was shown that optic nerve hyperintensity on diffusion-weighted imaging can be helpful in diagnosing indirect TON [31]. In another study, the diffusion tensor imaging technique showed no finding in the first week after injury, but by the second week, fractional anisotropy reduction became visible in the damaged eye and continued to be visible after one month [32]. These findings show that MRI is beneficial to detect changes in the later stages, but may not be as useful as CT in the early phases.

#### 3.3.5. Visual Evoked Potential

Although in many patients, the use of visual evoked potential (VEP) is not required to diagnose TON, it can be helpful in the diagnosis of suspicious cases and for determining the prognosis of vision. VEP has diagnostic value in patients who do not remember the time of nerve damage, patients with unreliable pupillary responses, and patients with bilateral TON. The chances of vision recovery are higher in patients with better responses to VEP [33–35]. Pattern reversal and flash VEP testing have been used to determine the rate of vision recovery. It has been reported that patients with a VEP amplitude of 50% in the damaged eye will have good vision recovery, while patients with an absent VEP will not have a good final vision [35–37]. Despite the diagnostic and predictive value of VEP, it has some limitations. For example, it is difficult to place a VEP device to the bedside of patients with multiple injuries in special wards. Patients may also have a concurrent brain injury, which can be mistaken for optic nerve damage, and this issue should not be dismissed [38].

#### 3.3.6. Optical Coherence Tomography

Several studies using optical coherence tomography (OCT) have shown retinal nerve fiber layer thinning in patients with TON. However, given that this finding may not be detected in the early stages and it is difficult to sit the patient up to do the OCT imaging, the value of OCT in the diagnosis of TON is reduced. OCT may be valuable in the long-term follow-up to show the progression of optic nerve injury over time [39–41].

#### 3.3.7. Doppler Sonography

Ultrasound Doppler has been employed in patients with TON to assess the hemodynamic indices of the central retinal artery (CRA). The reduction of peak systolic velocity (PSV), end-diastolic velocity (EDV), and time-average mean velocity (TAMX) have been reported in TON eyes [42]. This finding was confirmed by another study that showed reduction of PSV and EDV in the CRA of the injured eye [43].

#### 3.3.8. Visual Field

Although the visual acuity of patients with TON may be too poor which reduces the likelihood of achieving an acceptable outcome, automated visual field testing should be considered in all patients [21]. There is no specific visual field defect in patients with TON and any visual field impairment has been reported in patients; however, arcuate, central, and hemianopic field defects may be seen [29]. It is well documented that the visual field is severely compromised at baseline when it is compared with normal subjects, but a comparison of baseline visual field and long-term follow-up visit shows a significant improvement in visual field extension [44].

### 3.4. Treatment

#### 3.4.1. Medical Treatment of TON

A visual recovery rate of about 50% is expected following conservative management in indirect TON, where baseline VA plays the main role in the prediction of final visual outcome [22, 45–47]. To estimate the golden time of medical or surgical treatment, a longitudinal study by Kanamori et al. was performed to analyze the decrease in ganglion cell population and nerve fiber layer thickness following TON [48]. They reported that the decrease started two weeks after trauma and stopped changing after 20 weeks. Accordingly, it was suggested that the treatment should be started within 20 weeks following the incidence of TON.

The pharmacological rationale of using corticosteroids for TON arose from their benefits in the management of CNS injuries in animal models [13, 49]. According to these studies, it was hypothesized that steroids exert neuroprotective effects through their antioxidant properties [50]. Animal models of steroid efficacy for the treatment of TON, however, yielded inconclusive observations. Ohlsson et al. failed to show any effectiveness of steroid therapy on retinal axon and ganglion cell survival [51]. In another study, it was found that steroids exacerbated axonal loss following optic nerve damage in a dose-dependent fashion [52]. However, Lew et al. reported an improved optic nerve blood flow following high-dose corticosteroid therapy in 10 rabbits with experimental TON [53].

Considering human studies, the use of high-dose corticosteroids for TON was extrapolated from the National Acute Spinal Cord Injury Study (NASCIS) results [54]. Although the debate surrounding the NASCIS results has never come to a conclusion, a review by one of the investigators of the NASCIS supported the idea that steroids help in neurological salvage when administered within 8 hours of spinal cord injury [55].

Table 1 summarizes some studies on the effectiveness of steroids for the treatment of TON. The International Optic Nerve Trauma Study (IONTS) is the largest comparative study analyzing 133 cases of indirect TON treated with corticosteroids, surgical decompression of the optic nerve, and observation. Following adjustment for baseline VA, there were no significant differences between the three groups. Neither the dose nor the timing was associated with a higher probability of visual recovery. The improvement rates reported by other case series for steroid therapy in TON are comparable to IONTS, being around 50%.

In a placebo-controlled clinical trial, 31 eyes of 31 patients were divided into two management groups. Sixteen eyes received 250 mg intravenous methylprednisolone q6h for 3 days, followed by oral administration of 1 mg/kg prednisolone for 14 days; the other 15 eyes received a placebo [62]. The study confirmed previous observations that there is no difference in VA improvement between steroids and placebo in the treatment of TON.

In a pilot study in 2011, 7 cases with indirect TON received daily intravenous injections of 10,000 IU erythropoietin (EPO) for three days. They were compared to eight patients who received no specific treatment [65]. The final VA was significantly higher in the EPO group, suggesting a new safe and efficient treatment for patients with TON. In another case series, 18 eyes of 18 indirect TON patients received 20,000 IU EPO daily injections for three consecutive days [66]. This study reported an obvious effect of EPO on improvement of VA in patients with recent indirect TON.

In the Traumatic Optic Neuropathy Treatment Trial (TONTT), 120 patients underwent treatment with EPO, methylprednisolone, or observation [67]. Although color vision was reported to improve in the EPO group, improvement of VA was not significantly different between the three groups.

Complications of steroid and EPO treatment are rare; however, there is no definite evidence of any benefits of these drugs in terms of VA improvement in patients with TON. Thus, clinicians should be aware of the risk of severe side effects with an aggressive treatment protocol, especially when the effectiveness of the treatment is under debate. The Corticosteroid Randomization after Significant Head Injury (CRASH) study was terminated prematurely due to the increased rate of death in the high-dose corticosteroid group [68]. The Optic Neuritis Treatment Trial reported two cases of acute psychosis and acute pancreatitis in the steroid treatment group, both resolving without sequelae [69]. Transient hypotension has been reported in studies with EPO, which can be hazardous for patients with multiple trauma and unstable medical conditions [66].

#### 3.4.2. Surgical Treatment of TON

Regarding VA improvement TON cases, the effectiveness of surgical interventions is similar to that of medical therapies. However, surgical procedures may be superior to medical management since they can be performed according to pretreatment indications and be scheduled for selected patients. In addition, optimal surgical techniques have been investigated and updated through numerous studies performed by ophthalmologists, neurosurgeons, and head and neck surgeons.

Releasing the compression exerted by edema, hematoma, or fractured bone segments on the optic nerve is the rationale for surgery in TON. It can be indicated in the following situations: presence of bone segments or hematoma compressing the nerve in initial posttrauma images, poor response to initial medical treatment, evidence of optic nerve damage in preoperative VEP scan, or lack of evident damage to ocular tissues and intracranial optic nerve [70].

According to primary reports, it was recommended to perform surgery within 2 or 3 days following the trauma [71, 72]. However, in a recent meta-analysis, more than half of the patients treated surgically after seven days experienced visual improvement, and it was comparable with the early treatment group who underwent surgery within three days [73]. Accordingly, it may be recommended that in selected patients with appropriate indications, late surgical intervention is better than not intervening. Apparently, complete atrophy of the nerve, disruption of the intracranial portion of the ON, presence of carotid-cavernous fistula, and unstable systemic condition for general anesthesia should be considered as contraindications for any kind of surgical intervention [70].

The three main approaches performed for decompression of the optic nerve include medial transorbital and external ethmoidectomy, transcranial surgery, and endoscopic transnasal approach. Classic transorbital and transcranial approaches have been criticized for cosmetic problems; however, the main advantage of these procedures is the surgical view and wide optic nerve decompression. In contrast, transnasal approaches do not have cosmetic concerns, but relatively narrow decompression of the optic nerve may restrict optimal surgical outcomes. Between these two extremes of the surgical spectrum, some modification to surgical decompression of the optic nerve have been proposed. As a modification to the classic transcranial approach, the supraorbital approach may be performed through extradural unroofing of the optic canal via an endoscope [74, 75]. To overcome the limitations associated with the endonasal approach, total circumference decompression of the ON through a combined transorbital and transnasal approach is being developed [76].

Since the trend of optic nerve decompression has turned toward endoscopic approaches, Table 2 is presented to summarize some case series evaluating the efficacy of the procedure, in addition to presenting surgical indications and predictive pretreatment factors.

### 3.5. Prognosis

Direct and indirect TON have different prognoses. Direct TON causes severe, permanent visual impairment with slight probability for recovery [25], while visual recovery happens in 40–60% of cases with indirect TON that are managed conservatively. It is reported that in patients with indirect TON the visual function spared, if any, three months after trauma is maintained virtually forever in their life [44]. Baseline VA is the main predictor of the final outcome; therefore, initially poor VA is associated with limited or no visual recovery [5, 22, 45–47, 85]. Visual recovery and final VA may also be lower in cases with loss of consciousness, lack of visual recovery after 48 hours, absence of visual evoked responses, presence of blood within the posterior ethmoid cells, age over 40 years, lower grade of RAPD, optic canal fracture, and intraconal hematoma and hematoma along the optic nerve [5, 23, 36, 45, 61, 86–88].

### 3.6. Experimental Studies on TON

Several animal models have been proposed for studying direct and indirect TON. Optic nerve crush or transection have been used to simulate direct TON, while blast injury [40] and ultrasound-induced neuronal damage [89] have been developed for studying indirect TON. Although these methods have been effective to induce TON, they only look at the damage to optic nerve axons and retinal ganglion cells and do not consider extensive damage, such as brain injury associated with TON. Recently, a murine closed head traumatic brain injury model was proposed, studying indirect TON in the context of traumatic brain injury [90]. In this model, the optic nerve injury occurred at the optic canal level. The results of this model indicated neuroinflammation, gliosis, and axonal degeneration in the optic tract and major axonal targets of the optic nerve axons such as the superior colliculus and lateral geniculate thalamic nucleus. Since a majority of indirect TON cases occurs in patients with head injuries, this model may highlight the association between the TON and the complications of head injuries such as hemorrhage, blood-brain barrier disruption, brain edema, and increased intracranial pressure. Concomitant central nervous pathologies can interact with primary and secondary injury mechanisms to influence the progression of an optic nerve injury. The head trauma-based experimental models provide the opportunity to study the potential treatment options for nonvisual brain damages and their impact on optic nerve injuries [90].

## 4. Conclusions

TON is an uncommon vision-threatening disorder that should be considered in a patient with ocular or head trauma and decreased VA. Detection of an afferent pupillary defect in the presence of an intact globe and clear media strongly suggests TON, and neuroimaging must be performed in this clinical setting. Although there is no definitive treatment for TON, the use of EPO can be beneficial in some patients.

## Figures and Tables

**Table 1:** Summary of the studies on the effectiveness of steroids for the treatment of TON.

Study	Type of the study	Year	Number of eyes	Treatment protocol	Study result
Spoor et al. [56]	Comparative observational study	1990	22 eyes from 21 patients	13 patients were treated with intravenous megadose methylprednisolone, 8 patients were treated with high-dose dexamethasone	12 of 13 patients from methylprednisolone group and 7 of 9 eyes from dexamethasone group experienced improved visual function
					The difference between two group was not significant
Seiff [47]	Comparative observational study	1990	36	21 patients were acutely treated with high-dose intravenous dexamethasone and 15 were not	62% of the treated patients and 33% of the untreated patients showed visual improvement
					The difference was not statistically significant
Maureillo et al. [57]	Non-comparative observational study	1992	23	High-dose intravenous steroids were initiated in all patients	Nine of 16 patients who received steroids only showed significant improvement
				If vision did not improve significantly after 24 to 48 hours, decompression of the optic nerve was considered	
Chou et al. [45]	Comparative observational study	1996	58	23 patients were treated with intravenous dexamethasone or oral prednisolone; 25 patients underwent optic canal decompression in addition to medical treatment	13 of the 23 cases (57%) in the medical group had visual improvement
				10 cases were monitored without treatment	15 of 25 cases (60%) in the surgical group had visual improvement;
					none of the control patients showed any improvement in visual acuity
Levin et al. [22]	Comparative observational study	1999	133	9 patients received no treatment, 85 patients were treated with corticosteroids, and 33 patients underwent optic canal decompression	32% of the surgery group, 57% of the untreated group, and 52% of the steroid group showed visual improvement; there were no significant differences between any of the treatment groups
Wang et al. [23]	Comparative observational study	2001	61	25 patients treated with high-dose steroids, 7 patients underwent optic nerve decompression, 13 received no treatment, and others underwent facial surgery	There was no significant difference in the improvement of visual acuity in patients treated with surgical versus nonsurgical methods
Kitthaweesin [58]	Comparative observational study	2001	21	10 patients received dexamethasone intravenously, and 11 received intravenous methylprednisolone	70% of patients treated with dexamethasone, and 45% of patients treated with methylprednisolone experienced visual improvement
					There were no significant differences in the visual improvement between the two groups
Chuenkongkaew and Chirapapaisan [59]	Comparative observational study	2002	44	22 patients selected to receive intravenous high-dose dexamethasone, and 20 cases received megadose methylprednisolone	Visual improvement was shown in 37.5% of the dexamethasone group and 50% of the methylprednisolone group. There was no significant difference between the two groups
Yip et al. [60]	Comparative observational study	2002	21	9 patients were treated with intravenous methylprednisolone, and 12 patients were treated conservatively	Visual recovery was observed in 44.4% of eyes treated with methylprednisolone and in 33.3% treated conservatively, there were no differences in visual improvement between the two groups
Yang et al. [61]	Comparative observational study	2004	42	24 patients received treatment with megadose steroids combined with optic nerve decompression, and 18 with megadose methylprednisolone alone	Patients in a surgical group with an initial VA of NLP had a better visual improvement than those in nonsurgical group
Entezari et al. [62]	Randomized placebo-controlled clinical trial	2007	31	16 eyes received intravenous and oral corticosteroid, 15 eyes received normal saline as the placebo group	There was no difference in visual acuity improvement between intravenous high-dose corticosteroids and placebo
Lee et al. [15]	Comparative observational study	2010	116	75 patients received no acute treatment, and 41received steroids and/or surgery	Of the treated group, 24% and of the untreated group 20% showed improvement of VA
					There was no difference between the two groups in improvement of VA
Pokharel et al. [63]	Comparative observational study	2016	10	4 cases received intravenous methylprednisolone, and 6 cases were observed without steroid treatment	The visual recovery after intravenous steroid treatment was rapid and beneficial in cases with vision better than NPL
Sosin et al. [64]	Comparative observational study	2016	109	9 patients received intravenous corticosteroid, 62 patients underwent observation, 31 patients received surgical intervention, and the others underwent surgery and corticosteroid administration	Outcomes following corticosteroid administration and observation were comparable

**Table 2:** Summary of the case series evaluating the efficacy of the procedure, surgical indications, and predictive pretreatment factors.

Study	Year	The surgical approach to decompress ON	Number of patients	Conservative treatment	Indication for the surgery	Report of the efficacy of the surgery	Report of predictive factors
Yu et al. [77]	2018	Endoscopic endonasal	62	All cases were administered by methylprednisolone (20 mg/kg/day) and mouse-derived nerve growth factor	Patients with no VA improvement after intravenous treatment were recommended to endoscopic transethmosphenoid optic canal decompression	The overall visual acuity improvement rate after surgery was 54.84%	Patients with residual vision had better postoperative visual prognosis and benefited
							Treatment should still be recommended even for cases of delayed presentation
Gupta and Gadodia [78]	2018	Endoscopic endonasal	20	All cases received intravenous methylprednisolone for three days before the surgery	Radiologically evident bony fractured fragment impinging on optic nerve in the intracanalicular portion, or failure to improve after 48 h of steroid therapy, were indications for surgical intervention	80% of all patients benefited from the surgery	The patients who were operated within 72 h were most benefited
							All the patients with fractures were benefited
Xie et al. [79]	2017	Endoscopic endonasal	10 eyes from 5 patients	All the patients primarily treated with high-dose corticosteroids	All the patients underwent surgery due to poor response to medical therapy	Visual acuity improved in 30% of eyes	Surgical outcomes depend on both the timing of surgery and the severity of the damage manifested by initial visual acuity
Yan et al. [80]	2017	Endoscopic endonasal	1275	All patients received intravenous methylprednisolone of 500–1000 mg for the first 2 days and half dosage for the following days	The surgery was performed on patients whose VA was no more than 20/100 with no improvement after 4-5 days of conservative treatment	81.2% of patients experienced visual improvement	The presence and type of optic canal fractures may be a factor of visual prognosis
He et al. [81]	2016	Endoscopic endonasal	11	Not clarified	Indications for the surgery was optic nerve damage in preoperative VEP scans	Visual acuity improvement rate was 45.5%	The therapeutic effect relies on adequate decompression of the optic canal, timing of the surgery, and skillful surgical technique
Yang et al. [82]	2012	Endoscopic endonasal	96	Some patients treated with corticosteroid and the other not	Not clarified	The overall rate of effectiveness was 40.6%	No light perception, undergoing surgery 3 days after trauma, and hemorrhage within the ethmoid and/or sphenoid sinus were significantly associated with unrecovered visual acuity
Zuo et al. [83]	2009	Endoscopic endonasal	155	All patients received megadose steroid therapy	The surgery was performed after failure of megadose steroid therapy	The total effective rate was 44.5%	Residual vision after trauma and the interval between injury and surgery were significant prognostic factors
Li et al. [84]	2008	Endoscopic endonasal	176	All patients were treated with high-dose dexamethasone intravenously for three days, followed by 20 mg/day for three days and 10 mg/day for three days	Not clarified	The total vision improvement rate was 55%	Early surgery was an important prognostic factor for vision recovery

## Data Availability

The data supporting this review article are from previously reported studies which have been cited. The extracted information are available from Iman Ansari (imnansari71@gmail.com) upon request.
